# Gastrointestinal functional impairments and epilepsy: Searching the possible connection mechanisms

**DOI:** 10.1192/j.eurpsy.2021.1098

**Published:** 2021-08-13

**Authors:** R. Lefter, A. Ciobica, I.M. Balmus

**Affiliations:** 1 Center Of Biomedical Research, Romanian Academy, Iasi, Romania; 2 Department Of Biology, Faculty Of Biology, Alexandru Ioan Cuza University of Iasi, Iasi, Romania; 3 Department Of Biology, Academy of Romanian Scientists, Bucharest, Romania; 4 Department Of Interdisciplinary Research In Science, Alexandru Ioan Cuza University of Iasi, Iasi, Romania

**Keywords:** FGID, Epilepsy, irritable bowel syndrome, mechanisms

## Abstract

**Introduction:**

Epilepsy is one of the most common neurological disorders worldwide characterized by unpredictable and recurrent seizures, resulting from abnormal brain activity, accompanied by loss of consciousness and control of bowel or bladder function.

**Objectives:**

A higher risk of comorbid disorders in epilepsy has been reported for psychiatric affective conditions (i.e., depression and schizophrenia), sleep alterations, as well as some gastrointestinal disorders (inflammatory bowel disease and constipation), and lately there is an interest to determine and explain a putative association between functional gastrointestinal disorders (FGID) such as Irritable bowel syndrome (IBS) and epilepsy.

**Methods:**

In this way, we decided to review the current aspects of the gastrointestinal functional impairments and epilepsy by searching in the literature possible connection mechanisms.

**Results:**

A handful of studies have only recently reported an increased prevalence of IBS in epilepsy in children, in adults, and conversely a higher incidence of epilepsy in IBS patients at the populational level. Paroxysmal abdominal complaints resulting from seizure activity are present in the abdominal epilepsy syndrome and the link between constipation and seizures has been demonstrated in animal models. Currently, there is no data to directly address the cellular and molecular connections between epilepsy and FGID, but these would probably involve the bidirectional dysregulation of the brain-gut axis with increased afferent processing of visceral nociceptive signals and subsequent hyperalgesia.

**Conclusions:**

Thus, intestinal dysbiosis may play a role in triggering inflammatory and immune-related mechanisms reported in IBS manifestations and epilepsy, while vagal neuroimunomodulation issues are likely to be involved in both pathologies as well.

**Conflict of interest:**

The authors are currently supported by a Young Research Teams supporting research grant PN-III-P1-1.1-TE2016-1210, named ”Complex study on oxidative stress status, inflammatory processes and neurological manifestations correlations in irritable bowel synd

